# Removal of Reactive Dyes in Textile Effluents by Catalytic Ozonation Pursuing on-Site Effluent Recycling

**DOI:** 10.3390/molecules24152755

**Published:** 2019-07-29

**Authors:** Enling Hu, Songmin Shang, Ka-Lok Chiu

**Affiliations:** 1 State Key Laboratory of Silkworm Genome Biology, College of Textile and Garment, Southwest University, Chongqing 400715, China; 2 Institute of Textiles and Clothing, The Hong Kong Polytechnic University, Hong Kong, China

**Keywords:** effluent recycling, catalytic ozonation, textile dyeing, fabric color quality, magnetic collection

## Abstract

The textile wash-off process consumes substantial amounts of water, which generates large volumes of wastewater that pose potential pollution issues for the environment. In the present study, catalytic ozonation was applied to degrade residual dyes present in rinsing effluents from wash-off processes towards the aim of recycling the waste effluents. A magnetic catalyst was prepared for promoting dye degradation by catalytic ozonation. Via a hydrothermal reaction, highly magnetic manganese ferrite (MnFe_2_O_4_) particles were successfully loaded on carbon aerogel (CA) materials (MnFe_2_O_4_@CA). The results showed that the developed catalyst strikingly promoted the degradation of dye contaminants by catalytic ozonation, in terms of color removal and reduction of chemical oxidation demand (COD) in rinsing effluents. COD removal efficiency in catalytic ozonation was enhanced by 25% when compared with that achieved by ozonation alone under the same treatment conditions. Moreover, we confirmed that after catalytic ozonation, the rinsing effluents could be recycled to replace fresh water without any evident compromise in the color quality of fabrics. The color difference (Δ*E_cmc(2:1)_*) between fabrics treated with recycled effluents and water was not more than 1.0, suggesting that the fabrics treated with recycled effluents displayed acceptable color reproducibility. Although colorfastness and color evenness of fabrics treated with recycled effluents were slightly poorer than those of fabrics treated with water, they were still within the acceptable tolerance. Therefore, the present study validated that catalytic ozonation was a promising technology for saving water and wastewater elimination in textile dyeing. It provides a feasibility assessment of catalytic ozonation for recycling waste effluents to reduce water dependence in textile production. Furthermore, we show a new perspective in on-site recycling waste effluents by catalytic ozonation and enrich the knowledge on feasible approaches for water management in textile production.

## 1. Introduction

Textile dyeing is one of the most contaminative processes in textile wet processing [1]. Although new textile materials have been developed for wearing in different occasions, conventional cellulosic fibers still account for the largest proportion of fabric material in addition to synthetic fibers. The typical colorants for cellulosic fibers are reactive dyes which covalently bond with the hydroxyl groups in the cellulosic polymer molecules [2], thus leading to the excellent colorfastness of colored products [3]. Nevertheless, the most acute problem in the coloration of cellulose with reactive dyes is the relatively low utilization efficiency of reactive dyes [4]. Some dyes hydrolyze before reacting with cellulose and generally attach to fibers by physical absorption and/or adsorption. These loose dyes must be extensively cleaned, or they will gradually leach during service after the textile products are fabricated, causing deficient product quality, contaminating other items and even putting users under heath risks [5].

Consequently, the wash-off process, in which fabrics are extensively rinsed after dyeing, is an indispensable treatment. According to the water footprint calculation, the water used in the wash-off process represents the largest proportion of the total water consumption in the entire textile manufacturing industry from raw fibers to final products [6]. Multiple water baths are used to rinse the dyed fabric in sequence, repeating the “rinsing-discharging” cycle several time until fabrics are thoroughly cleaned. These spent rinsing effluents, which contain dyes and other chemical additives, are highly toxic to both living species and the aqueous environment [7]. If discharged without appropriate treatments, they can severely deteriorate the aquatic ecosystem [8], ultimately resulting in harmful effects on humans [9]. Therefore, water savings and wastewater treatment in the textile manufacturing industry have attracted growing attention [10].

In the past three years, the environmental regulations in China have become more restrictive than in the past. New policies to regulate wastewater management in the textile industry have been promulgated. Thus, the demand for sustainable manufacturing methods for textile products to save water and reduce wastewater generation is urgent [11]. With this in mind, some feasible processes have been developed for effluent reclamation for reuse, including adsorption [12], electro-coagulation [13], ozonation [14], and some other comprehensive technologies [8,15]. Ozonation, in which ozone gas is applied as the oxidant for degradation of contaminants, has various advantages for industrial applications [16]. Especially when assisted with catalysts, the degradation capacity of ozone can be further enhanced [17]. Therefore, as extensions of two pioneer studies that date back to 2007 [18,19], the reuse of waste dyeing effluent containing high content of residual dyes was successfully realized in successive dyeing [14]. In general, a typical batch-wise dyeing process could be roughly divided into three stages in sequence, including exhaustion of dyes, fixation of dyes and wash-off. Although sometimes the former two steps are implemented together in one single water bath, the wash-off process composing several rinsing steps must be performed separately with multiple water baths [3]. In general, the volume of spent rinsing effluents is much larger than that of spent dyeing effluents. The total volume of waste rinsing effluents could be four to seven times that from dyeing, depending on the color depth required in dyeing. Although rinsing effluents are much less contaminative than dyeing effluents, the large volume of water required and effluents discharged are concerning because saving water is important in textile manufacturing [20].

Some studies have focused on reduction of water consumption in the textile wash-off process. Special chemicals have been developed to improve rinsing efficiency, such as dosing functional polymers that can inhibit dye transfer [21] or adding micro-absorbents [22] in rinsing baths to remove residual dyes. These technologies mainly focus on reducing dye concentration in the rinsing bath to improve its cleaning capacity and reducing the number of rinsing baths to save water. However, it was worth noting that these “add-on” methods may further increase the pollution levels in wastewater, because these macromolecular chemicals dosed in rinsing baths lead to remarkably increased chemical oxygen demand (COD) in effluents. In addition, all original organics in effluents contributing to pollution only changed their accumulation/dispersion status, and there is no reduction in mass. Notwithstanding the fact they drastically improve the cleaning capacity of rinsing baths and potentially decrease water consumption, these benefits are accompanied with deficient water quality of the rinsing effluents. This predicament has redirected the authors to seek an alternative approach that applies a “cut-off” principle in reduction of water consumption.

Instead of adding macromolecular chemicals, ozonation treatment for wastewater applies gaseous ozone is added to remove organics, eliminating secondary pollutants arising from the addition of external macromolecular chemicals. In our previous study, we proved that ozonation exhibits excellent performance in dye elimination in terms of decolorization and COD removal [14]. In general, the dye concentration in rinsing effluents is far less than that in the dyeing effluents which contains as high as 40% of the total dyes dosed in the dyeing process. Considering that dyeing effluents can even be recycled after catalytic ozonation [14], we expected that the less contaminative rinsing effluents may also be recyclable after ozonation treatment.

Considering that the concentration of dyes striped from dyed fabric plays an important role in rinsing results, effective elimination of dyes presented in waste effluents should be beneficial for to recycling rinsing effluents. In theory, complete removal of dyes presented in waste effluents should restore the rinsing capacity of the waste effluents. However, although ozonation treatment can decolorize dye-containing effluents, it is difficult to convert organic dyes into inorganic salts completely [23]. Because of the limitation of selective oxidation by ozonation, chemically inert products from semi-degradation of the dyes may accumulate. Treatment efficiency would therefore be reduced gradually, in terms of COD reduction. Therefore, to enhance the degradation capability of ozonation treatment, a novel catalyst was developed for improved degradation of dyes by total mineralization, in which all organics were oxidized into inorganic salts.

Accordingly, in the present study, manganese ferrite (MnFe_2_O_4_) particles were loaded onto a carbon aerogel (CA), forming MnFe_2_O_4_@CA, as the ozonation catalyst. In contrast to our previous studies, in this study we utilized much finer particles of CA as the base support for preparation of MnFe_2_O_4_@CA, because a finer size can enhance the catalytic activities of numerous non-metal catalysts [24]. However, a finer particle size of the catalyst can lead to issues in the separation of solid catalyst particles from the aqueous phase after treatment. Therefore, highly magnetic MnFe_2_O_4_ nanoparticles were used to facilitate separation of fine catalyst particles from effluents using external magnetic fields [25]. In addition, MnFe_2_O_4_ exerts high catalytic activity during ozonation [26]. The bimetallic oxide has multivalent states and can promote electron transfer during the redox reactions of catalytic ozonation [27]. Furthermore, MnFe_2_O_4_@CA can be easily fabricated at relatively low temperature via hydrothermal methods [28].

Apart from the technical aspects, ozonation treatment has some additional advantages from the point of view of engineering. Specifically, ozonation devices are generally small and versatile; thus, they can be compactly integrated with existing dyeing machinery. This characteristic makes ozonation technology a unique and more promising in on-site treatment of waste effluents. If an ozonation reactor is connected to a dyeing machine in a dyehouse, spent effluents can be treated on-site rather than remotely in the central wastewater treatment plant. This on-site treatment mode of ozonation technology can shorten the reclamation period and facilitate quality control of treated effluents before recycling. By contrast, this on-site treatment mode is generally not applicable to other advanced oxidation processes. For example, photolysis, which is an important research topic follows the “cut-off” principle and depends on irradiation of wastewater by UV light. For saturated and thorough irradiation, the ultra violet lamps should be placed under water, which is a limitation because the photolysis system can leak because of the hydraulic pressure under water. In addition, ozonation devices are more compatible with existing overflow dyeing machines which are universally equipped in actual industrial production lines. Because the majority of key components and the connecting system are similar to those integrated in the existing dyeing machinery, the existing dyeing machinery does not have to be upgraded to integrate the ozonation system, thus making this technology more feasible than others.

**Figure 1:**
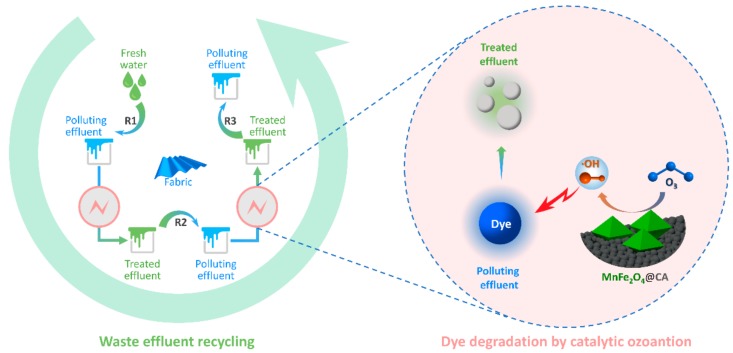
Recycling of spent rinsing effluents by catalytic ozonation.

In this study, we assess the color quality of fabrics rinsed with recycled waste rinsing effluents. The color specifications, such as color differences, and colorfastness should not be obviously sacrificed when compared with those of fabrics rinsed with fresh water. Thus, the rinsing capacity of recycled effluents must be equivalent to that of fresh water. Therefore, catalytic ozonation was applied for reclamation of the waste effluents before recycling and reuse. A schematic diagram of effluent reclamation for recycling is shown in Figure 1. Firstly, water was used in the first rinsing step (R1) and then the resultant effluents were restored rather than discharged as wastewater. For recycling, the collected spent effluents were then treated by ozonation treatment to eliminate color contaminants before being reused in the second rinsing step (R2). Likewise, the “treatment-reuse” cycle was repeated in a third rinsing step (R3), and the wash-off process for fabrics was accomplished. During ozonation treatment, to tackle the shortcoming of ozonation of selective oxidation, MnFe_2_O_4_@CA catalyst was adopted to enhance elimination of organic contaminants. Dissolved ozone in the aqueous solution was expected to decompose catalytically into hydroxyl radicals (OH), which have higher redox potential than ozone itself and could degrade organic pollutants non-selectively. Accordingly, residual dyes in the effluents were converted into inorganic salts extensively. Owning to the strikingly improved degradation of residual dyes, the treated effluents may be recycled on-site in textile wash-off process, thus saving water in the textile wash-off process.

A review of previous literature showed that reducing water consumption in textile wash-off process has hardly been studied. In the present study, we hope to lend support to the efforts made to water saving in textile manufacturing. The results of the study may broaden applications of ozonation technology in textile wastewater control.

## 2. Results and Discussion

### 2.1. Characterization of Catalyst Materials

**Figure 2:**
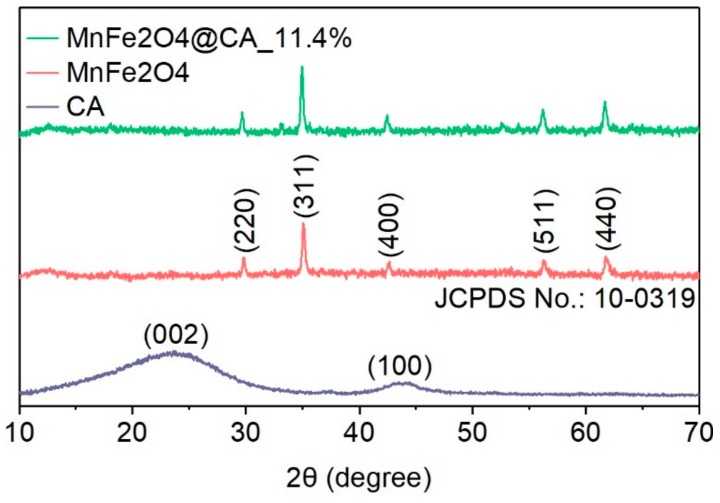
XRD pattern of CA and MnFe_2_O_4_@CA.

Figure 2 shows the X-ray diffraction (XRD) patterns of MnFe_2_O_4_@CA and its pure components. Like most carbon materials, two wide peaks appeared at 23° and 43° for CA. They could be indexed to the planes of (002) and (100) arising from amorphous carbon [29].

For MnFe_2_O_4_@CA, the peaks located at 30.2, 35.5, 43.1, 57.0 and 62.6 could be associated with diffractions from (220), (311), (400), (511) and (440) planes of the cubic spinel structure of MnFe_2_O_4_, according to JCPDS No.10-0319 [30]. In addition, the sharp-pointed peaks from MnFe_2_O_4_@CA confirmed that the MnFe_2_O_4_ nanoparticles possessed high crystallinity.

**Figure 3:**
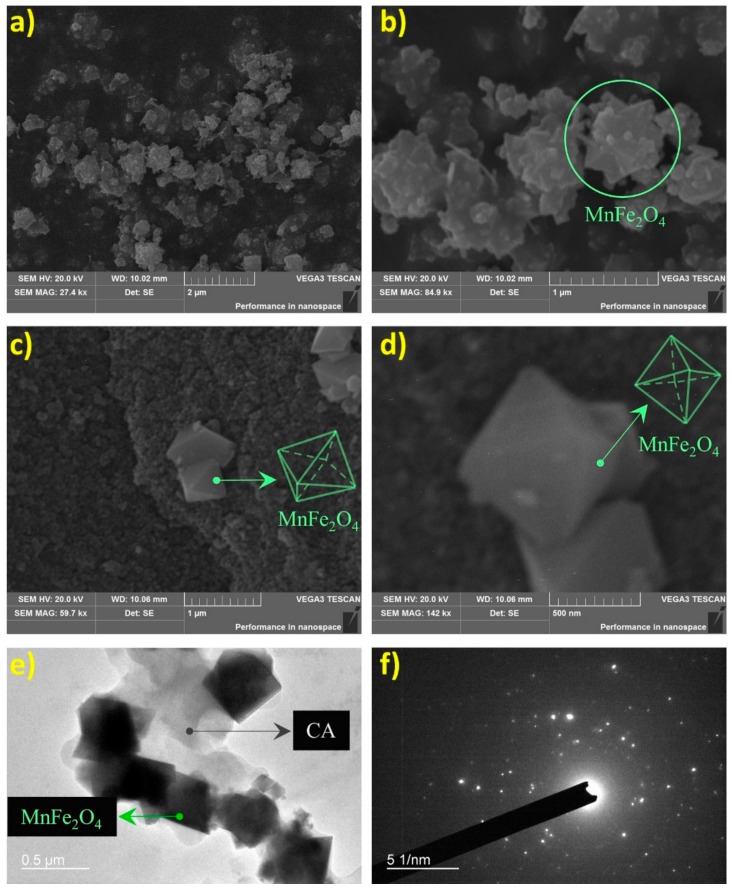
SEM images MnFe_2_O_4_ (**a**,**b**) and MnFe_2_O_4_@CA (**c**,**d**), and TEM (**e**) and SEAD (**f**) of MnFe_2_O_4_@CA.

Scanning electron microscopy (SEM) images of MnFe_2_O_4_@CA and its components are shown in Figure 3a–d. It was evident that the shapes of MnFe_2_O_4_ prepared alone and prepared in-situ over CA were quite different. Pure MnFe_2_O_4_ prepared in the absence of CA was irregularly cuboid (Figure 3a,b). Multiple edges can be found on the surface of MnFe_2_O_4_, similar to the shape of MnFe_2_O_4_ prepared by a similar approach [31]. Nevertheless, the shape of MnFe_2_O_4_ prepared in-situ over CA was much more regular. A typical octahedral nanostructure could be observed (Figure 3c,d). The inconsistent shape may be caused by the CA substrate dispersed in the solution which could have impacted the growth behavior of MnFe_2_O_4_. This inconsistent shape also found in some crystals prepared over base substrates [32]. The crystal shape of MnFe_2_O_4_ has been studied previously as a function of calcination temperature, at temperatures ranging from 900–1200 °C [33]. The present nanooctahedral shape obtained was in line with that prepared after calcination at 1100 °C.

Figure 3e shows transmission electron microscopy (TEM) images of MnFe_2_O_4_@CA. Particles with right angles can be identified as MnFe_2_O_4_ according to the shape illustrated in Figure 3c,d. The selected area electron diffraction (SEAD) pattern of MnFe_2_O_4_ can be found in Figure 3f, which was consist with the results of a previous study [34].

**Figure 4:**
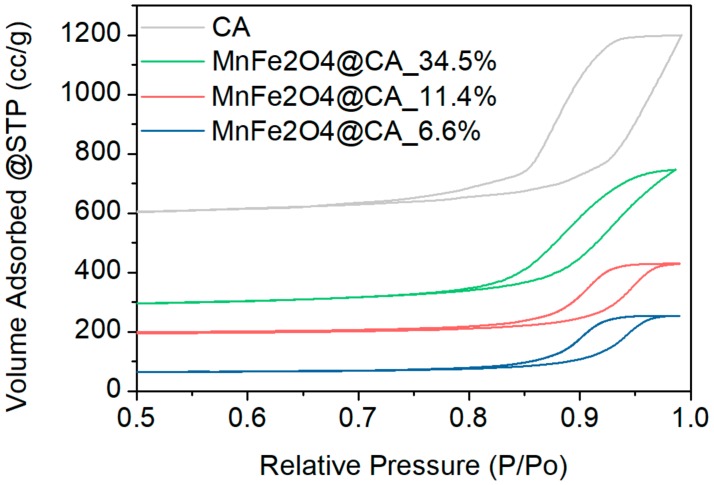
Nitrogen adsorption-desorption isotherms of catalyst materials.

The adsorption and desorption performances of the catalyst materials towards N_2_ are demonstrated in Figure 4. Both CA and MnFe_2_O_4_@CA showed an obvious hysteresis loop arising from capillary condensation at higher relative pressure. The type IV isotherm patterns confirmed the mesoporosity and macroporosity of both materials [35]. This maybe because CA was composed from numerous carbon nanoparticles which agglomerated, leaving numerous mesopores and macropores inside the material substrate.

The Brunauer–Emmett–Teller (BET) surface area and pore specifications are listed in Table 1. It was evident that the specific surface area was drastically decreased when the catalyst was decorated with more MnFe_2_O_4_ nanooctahedrons. When 11.4% of MnFe_2_O_4_ was loaded over CA, the specific area of the material dropped to around 380 m^2^/g, corresponding to a reduction of 35%. This could be because of the sacrifice of micro- and macro-pores of the CA substrate during the hydrothermal reaction for hosting MnFe_2_O_4_. The assumption can be proved by the variation trend of pore volume demonstrated in Table 1.

**Figure 5:**
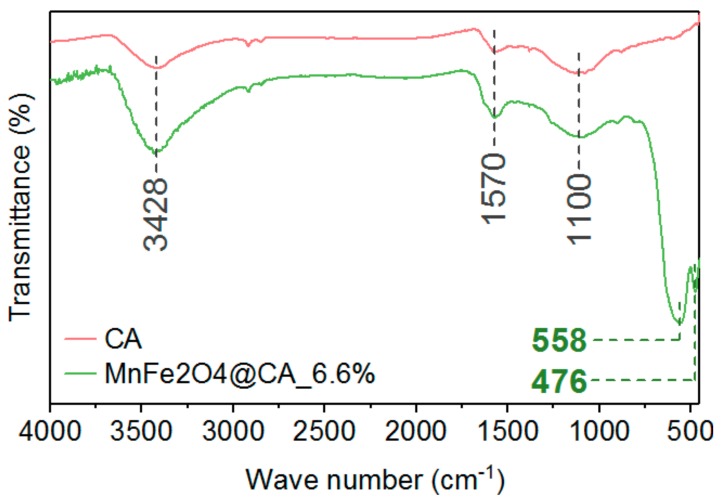
FTIR of MnFe_2_O_4_ and MnFe_2_O_4_@CA.

Figure 5 shows the Fourier-transform infrared spectroscopy (FTIR) spectra of CA and MnFe_2_O_4_@CA. The boarder band located at 3428 cm^−1^ corresponds to stretching and bending vibration of the hydroxyl group from adsorbed water or free water molecules present in the sample [36]. The weaker peak at 1570 cm^−1^ could be attributed to the H–O–H stretching vibration of absorbed water [37]. There was another typical peak located at 1110 cm^−1^, which could be assigned to the hydroxyl groups bonded on metal [38]. In addition to the above three typical peaks appearing in CA, there are two stronger peaks at 558 cm^−1^ and 475 cm^−1^. The former one corresponded to the intrinsic stretching vibration of manganese-oxygen (Mn-O) bonds, which was caused by the MnFe_2_O_4_ of octahedral shape [39]; the latter could be attributed to the intrinsic vibration of metal-oxygen (Mn-O and Fe-O) bonds at octahedral sites from MnFe_2_O_4_ [40], confirming MnFe_2_O_4_ prepared in-situ over CA had spinel structure [41]. These results confirmed the presence of octahedral MnFe_2_O_4_ over CA, which was in line with the evidence obtained in Figure 3.

**Figure 6:**
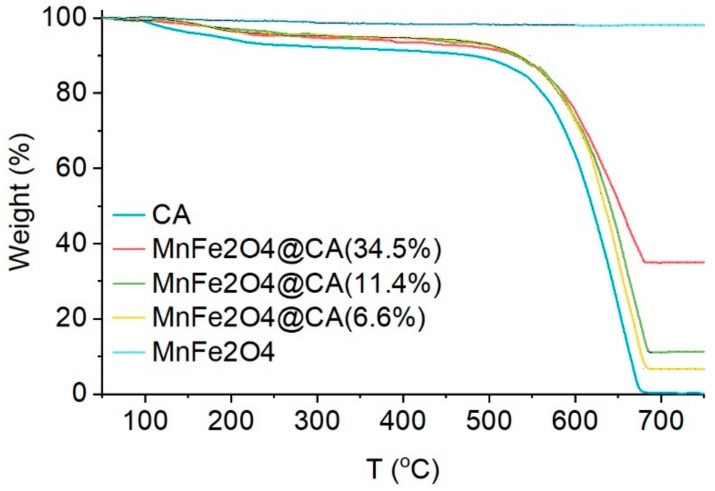
TGA of CA and MnFe_2_O_4_@CA.

Figure 6 shows the thermogravimetric analysis (TGA) curves of the different materials in air. The weight of CA started to drop when the temperature increased beyond 100 °C, maybe because of volatilization of adsorbed water, free water, and/or residual unreacted reactants present in the CA substrate. A similar phenomenon could be observed on MnFe_2_O_4_@CA, except the weight loss of MnFe_2_O_4_@CA was less than that of CA. Since the hydrothermal reactions for loading MnFe_2_O_4_ over CA were performed under high pressure at high temperature, we could assume that the difference in weight loss between pure CA and MnFe_2_O_4_@CA stemmed from the removal of unreacted reactants, which were previously used to prepare CA substrate, present in the final CA. The weight of materials containing CA declined rapidly when the temperature was further raised. This was because solid CA was oxidized by air to release CO_2_. During the entire TGA ranging from 50 to750 °C, no weight loss was observed over MnFe_2_O_4_, confirming its excellent thermal stability in air. In addition, loading of MnFe_2_O_4_ on CA had no change on the thermal properties of CA substrate, as the decomposition behaviors of CA and MnFe_2_O_4_@CA were almost the same.

### 2.2. Degradation of Reactive Dyes in Spent Wash-Off Effluents

As mentioned earlier, ozonation alone is generally incapable of completely degrading the organic contaminants in effluent. Thus the addition of catalyst to promote degradation was necessary. To confirm the behavior of MnFe_2_O_4_@CA in dye degradation in effluents, residual color and COD present in effluents treated at different time intervals were measured. The spent effluent, E_r1_, from R1 was collected as the probe water for reclamation.

#### 2.2.1. Color Removal in the Spent Rinsing Effluent (E_r1_)

Removal of color in spent rinsing effluents is critical before effluent recycling. The rinsing capacity is highly dependent on the concentration of colorants accumulated in the rinsing effluent. Stripping dyes from fabrics effectively would be difficult if the residual dyes were not eliminated sufficiently, because stripping of dyes by rinsing was significantly affected by the diffusion of loose dyes from fabric to the aqueous bath. Thus, in conventional process, the rinsing effluents which contain substantial unfixed loose dyes are discharged and replaced with water to sustain the rinsing efficiency in the wash-off process. Considering the adverse roles of residual dyes in spent effluents, color/dye elimination of effluents before recycling is important.

**Figure 7:**
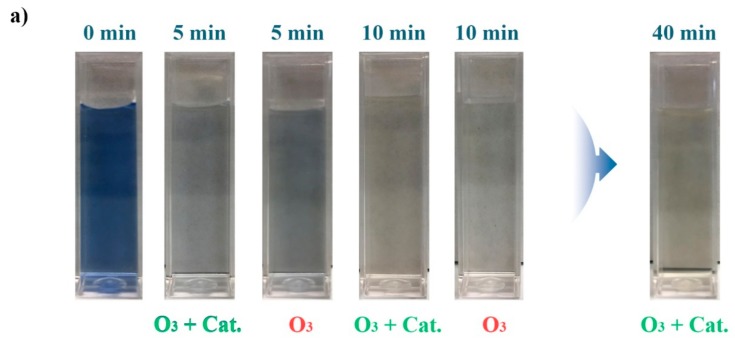

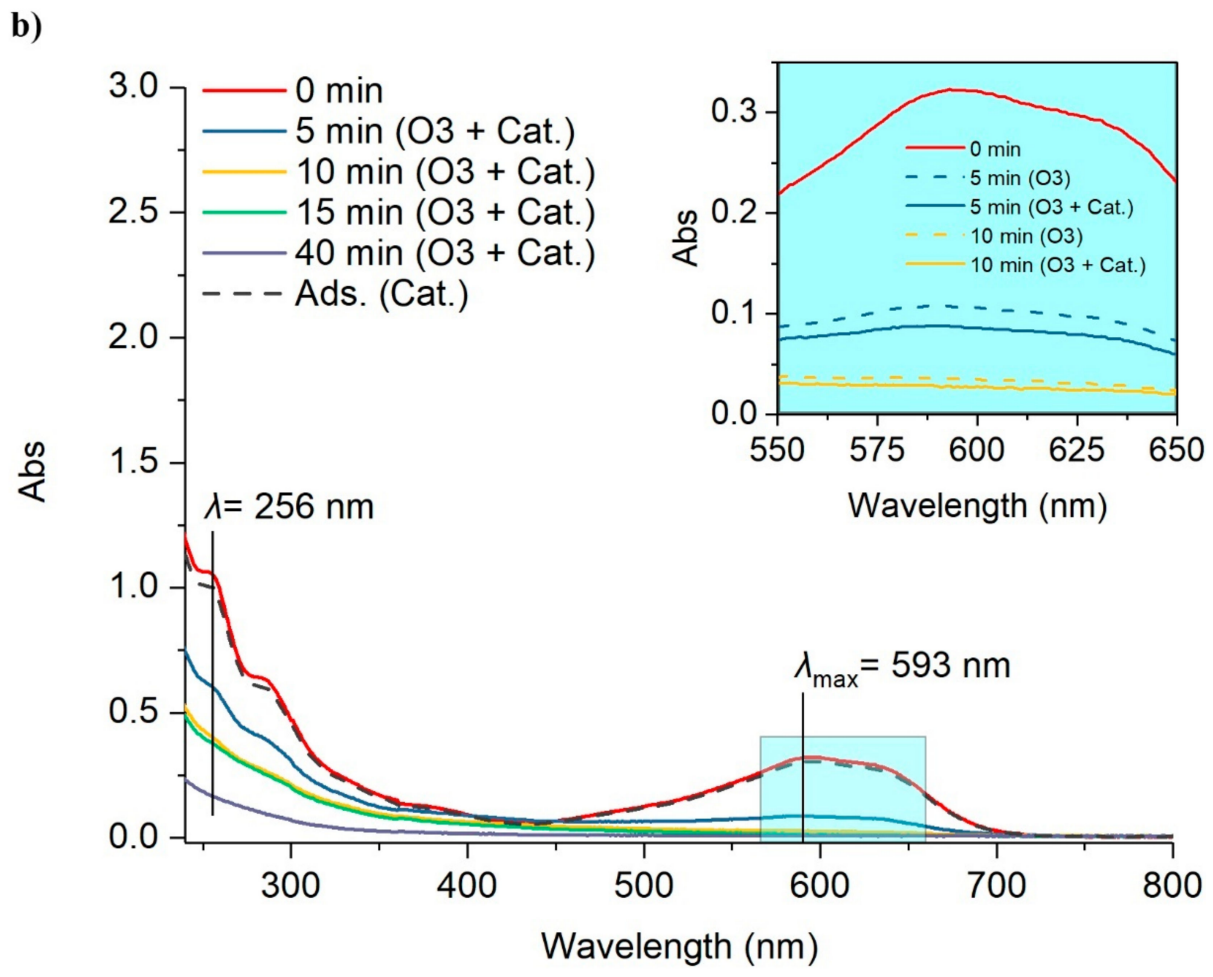
Color of E_r1_ treated by various processes: digital photos (**a**) and UV-vis absorbance (**b**) of E_r1_ after different treatment time.

In this study, effluent E_r1_ from the first rinsing bath was collected as the probe effluent to examine color removal. The color evolution of E_r1_ reclaimed by ozonation alone (O_3_) without catalysts and catalytic ozonation (O_3_ + Cat.) with MnFe_2_O_4_@CA (11.4% MnFe_2_O_4_ loaded on CA) is displayed in Figure 7a. At the beginning, the effluent was blue because of the presence of RB19; after treatment with ozonation alone or catalytic ozonation, color fading occurred in the effluents as time increased. After 10 min, effluent treated by catalytic ozonation had completely decolorized completely, whereas the effluent treated by ozonation was still bluish. The full UV-vis spectra of the selected effluents are illustrated in Figure 7b. Mainly three typical adsorption peaks were present within the range of 240–800 nm. The maximum adsorption peak at a wavelength of 593 nm (λ_max_ = 593 nm) and the smaller peak at a wavelength of 256 nm (λ = 256 nm) could be assigned to the anthraquinone chromophore present in RB19 molecule [42]. As the catalytic ozonation time increased, the absorbance of effluents rapidly decreased. After 10 min, the absorbance at λ_max_ = 593 nm was ultralow, suggesting that there were almost no dyes with intact chromophore present in effluents. This could lead to the assumption that the anthraquinone structure was degraded in catalytic ozonation, which could be supported by the peak at λ = 256 nm also vanishing. In addition, it was observed that color removal by adsorption of the catalyst was minimal. After 60 min of adsorption, the absorbance of the effluent was almost unchanged, suggesting dyes captured by the catalyst via direct adsorption could be ignored. In Figure 7 (inset), color removal after catalytic ozonation was compared with that after ozonation alone. In the first 10 min, most of the color was drastically removed. It was obvious that catalytic ozonation with MnFe_2_O_4_@CA was more efficient than ozonation alone without catalysts in color elimination. This suggested that there were more dyes present in the effluent treated by ozonation alone after the same treatment time, although the difference was not significant. This could be ascribed to the catalytic roles of MnFe_2_O_4_@CA. The presence of the catalyst substantially promoted generation of OH, which is more powerful than molecular ozone in oxidation of organics.

#### 2.2.2. COD Elimination in Spent Rinsing Effluents (E_r1_)

In addition to color removal, COD removal was also examined because the evolution of COD removal is generally different from that of color removal. In removal of color by physical adsorption, COD is generally reduced along with color at the same rate because all the organic colorants contributing to color also increased COD in effluents during adsorption. Thus, as color is removed via adsorption of colorants, COD is reduced simultaneously at a similar rate. However, in ozonation treatment, the removal of color and COD are desynchronized. In ozonation treatment, 100% removal of color only required the cleavage of all chromophores in dye molecules, which hardly contributes to COD removal, depending on the molecular weight and chemical activity of the probe dyes. Thus COD evaluation was necessary to understand the impact of colorless organics in effluents, as it precisely measures the mass of organics quantitatively [43].

**Figure 8:**
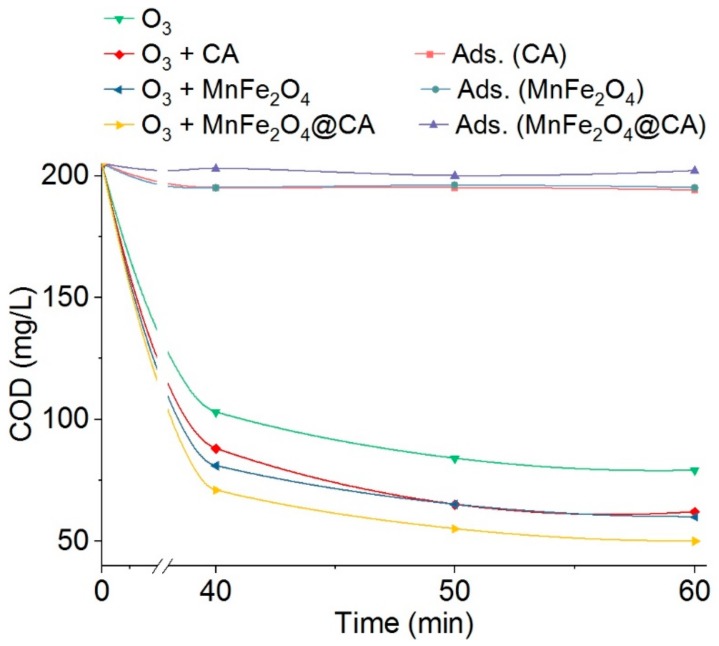
Evolution of COD of E_r1_ in different processes.

Figure 8 displays the residual COD present in effluents. Different from color removal shown in Figure 7, COD removal was much slower. After only 15 min of ozonation treatment, almost 100% of color was removed; however, as long as 40 min of treatments only ensured no more than 65% (reduced from 205 mg/L to as low as 71 mg/L) removal of COD in catalytic ozonation with MnFe_2_O_4_@CA.

In effluents, the only contributor of color are the whole chromophores from dye molecules, whereas contributors for COD are not only the chromophores and other groups from the entire dye molecule but also incompleted degradation products from dye oxidation by ozone. Consequently 100% COD removal requires conversion of organics into inorganics rather than simple cleavage of all anthraquinone structures. Accordingly, COD removal may also occur with color removal, but its reduction percentage may be marginal when compared with that of color removal. Therefore, 100% removal of color was not adequate in ozonation treatment, as there may be still substantial organic pollutants which may impact the rinsing ability of recycled effluents.

In addition to the difference of color and COD removal percentage, it was observed that the role of catalysts was more prominent in reducing COD. Both pure CA and MnFe_2_O_4_ could radically promote COD reduction compared with ozonation alone without any catalysts. After 40 min treatment, CA and MnFe_2_O_4_ enhanced COD removal by 15 mg/L and 22 mg/L, respectively, in catalytic ozonation. Since adsorption hardly decreased COD after 60 min, it could be inferred that the enhanced removal was attributed to the accelerated oxidation reactions. The universal recognition for the phenomenon was that the improved dye degradation is a result of generation of ·OH from ozone decomposition [44]. Moreover, when CA was incorporated MnFe_2_O_4_ to form MnFe_2_O_4_@CA, the catalytic performance was further improved. Treatment for 60 min only removed 60% (202 mg/mL reduced to 80 mg/mL) of the initial COD present in effluents by ozonation alone, whereas this removal was enhanced to 75% (202 mg/mL reduced to 50 mg/mL) by catalytic ozonation with MnFe_2_O_4_@CA. The removal efficiency was relatively enhanced by 25% (60% increased to 75%). In contrast, ozonation alone removed COD by 15% only, and the remaining 80 mg/L of COD was present in the treated effluent. Similar to color removal by adsorption, COD removal by adsorption between CA, MnFe_2_O_4_ and MnFe_2_O_4_@CA was hardly different and were all no more than 5%. Therefore the substantial drop in COD was a result of chemical oxidation which caused by MnFe_2_O_4_@CA. This was possibly because the multivalence oxidation states of MnFe_2_O_4_, combined with nonmetal carbon materials promoted electron transfer over the interface/surface of catalysts [45,46].

#### 2.2.3. Water Quality of Effluents in Recycling

**Figure 9:**
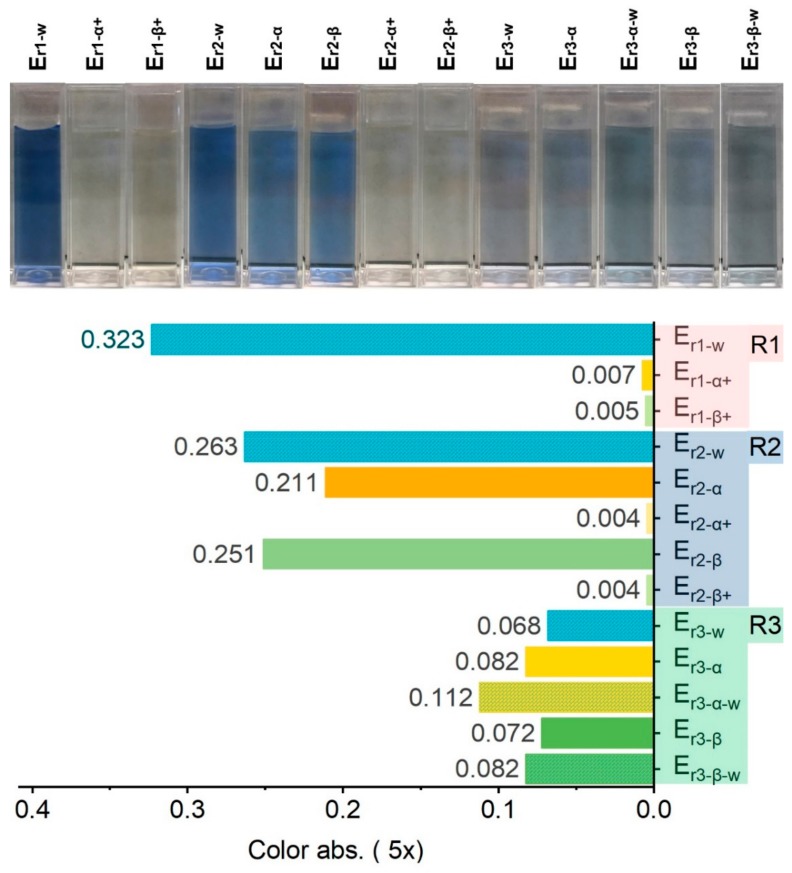
Color evolution of selected effluents.

Catalytic ozonation can radically remove color and eliminate COD in spent effluents. However, it was still unclear whether the quality of the effluent reclaimed was good enough for recycling. Consequently, effluent E_r1_, which was collected from R1 was reclaimed using ozonation with and without MnFe_2_O_4_@CA (11.4% MnFe_2_O_4_ loaded on CA) for 60 min. The reclaimed effluents E_r1−*α*+_ and E_r1−β+_ were then reused in R2. The rinsing-reclamation process was performed twice successively to finish the wash-off process which contained three rinsing steps in total. To measure the water quality of all effluents, color densities of the selected effluents are compared in Figure 9.

After R1, the resulting E_r1−w_ possessed the deepest color among all selected effluents because numerous unfixed dyes still existed, which physically attached on fabrics right after dye fixation and were diffused into the rinsing effluent. In addition, water used in R1 was favorable for stripping loose dyes from fabrics when compared with recycled effluents. Thereafter, E_r1−w_ was totally decolorized by ozonation treatment regardless of the catalysts use. Both effluents E_r1−α+_ and E_r1−β+_ turned to colorless irrespective of the treatment applied because ozone is an excellent remover of color. Fifteen minutes were adequate to convert E_r1−w_ into colorless by ozonation treatment (Figure 7). After R2, the highest color depth was observed in effluent E_r2−w_ because the reclaimed effluents, after either ozonation alone or catalytic ozonation, were still not as competent as water to clean fabrics. This suggested that although color removal was successfully accomplished in the reclaimed effluents, the residual colorless pollutants from uncompleted degradation of dyes in effluents should be considered carefully. Moreover, it was evident that E_r1−β+_ was more efficient than E_r1−α+_ in cleaning of fabrics, as the color of E_r2−β_ was higher than that of E_r2−α_. This suggested there were some unfixed dyes still present in F1_−r2_ and F2_−r2_. In addition, although catalytic ozonation could not fully renew the rising capacity of the spent effluents to make them similar to that of water, it was more efficient than ozonation alone.

Thereafter, E_r2−α_ and E_r2−β_ were further treated by ozonation treatments to form E_r2−α+_ and E_r2−β+_, which became colorless and transparent again, before being recycled for R3. According to Figure 9, color absorbance of E_r3−α_ and E_r3−β_ were almost identical. Considering that the difference of absorbance between E_r2−α_ and E_r2−*β*_ was remarkable, it could be inferred that there was still some loose color on F1_−r2_ and F2_−r2_ that had not been removed by E_r2−α_ during R2. Moreover, color absorbance of E_r3−α−w_ was significantly higher than that of E_r3−α_, confirming that cleaning of fabrics F1_−r1_ and F2_−r1_ by E_r1−α+_ was insufficient because some more unfixed dyes were cleared from fabric F2_−r3_ by water in R3. By contrast, there was only a small difference between E_r3−β−w_ and E_r3−β_ in color, confirming that the rinsing capacity of E_r2−β_ was better than that of E_r2−α_, and almost equivalent to that of water.

**Figure 10:**
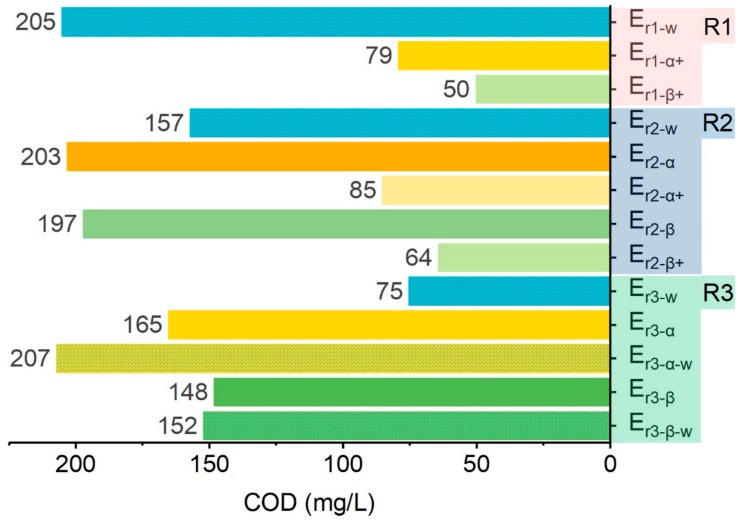
COD evolution of selected effluents.

Different from color evolution of effluents for which the absorbance was “reset” to zero after ozonation treatment regardless of the presence of catalysts, COD slowly accumulated with the recycling times (Figure 10). Although COD levels dropped after ozonation treatment, they did not become zero. This phenomenon originated from the fact that removal of color was much easier than removal of COD. During the treatment, by-products from primary degradation of dyes, together with the newly unfixed dyes diffused from fabrics, led to slowly increased COD. Nevertheless, the final COD of effluents from R3 was fairly controlled because ozonation treatment degraded organics. Although the effluents had been recycled twice, COD in effluents E_r2−α_ and E_r2−β_ was no more than that in E_r1−w_. It could be validated that, as an effective treatment following the “cut-down” principle, reclamation of effluents via catalytic ozonation not only led to water saving in wash-off process but also controlled pollution in wastewater effectively, provided that the color quality of fabrics was not downgraded beyond acceptable tolerance.

### 2.3. Determination of Fabric Color Quality

Determination of color and COD in effluents was only a method to understand the degradation behavior of ozonation treatment. It was more important to confirm the feasibility of catalytic ozonation in reclamation of spent effluent for recycling, which was the ultimate purpose of adoption of catalytic ozonation. Consequently, to understand the influence of recycled effluents on the color quality of fabrics, color reproducibility, colorfastness and color leveling property were determined to assess the integrity of fabrics in terms of color specifications.

#### 2.3.1. Color Reproducibility

To assess color reproducibility, the color differences in fabrics dyed with three reactive dyes and rinsed with various rinsing effluents were evaluated using the CIELAB color space model [47]. For fabrics dyed with RR239 and RY176, the ozonation time for reclamation of the spent rinsing effluents before recycling was set at 40 min, while other treatment conditions were unchanged.

**Figure 11:**
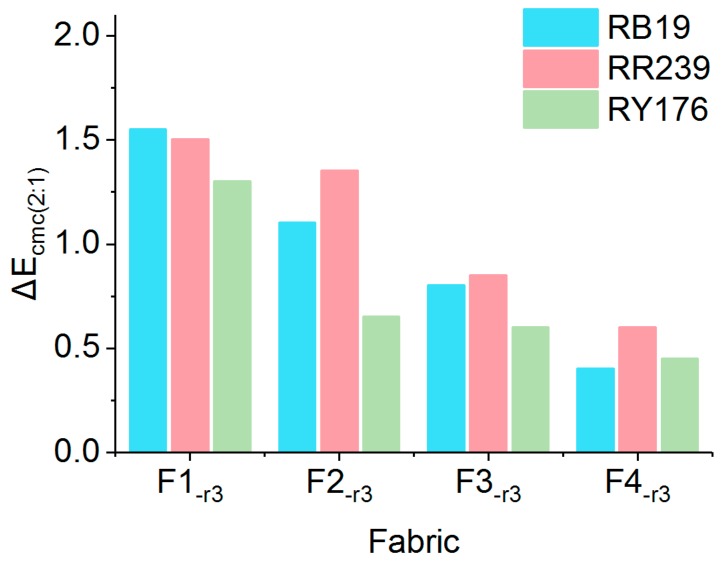
Color difference of fabrics dyed with different dyes.

The consistency of color between fabrics treated by effluents and water was measured by color difference (Δ*E_cmc(2:1)_*) which is the most adopted objective specification to identify any deviation of color between two fabrics. A smaller value of Δ*E_cmc(2:1)_* indicates there is smaller color deviation between fabrics. As is demonstrated in Figure 11, RB19-dyed fabrics rinsed with different rinsing medium were different in color consistency compared with the reference fabric sample (F5_−r3_) for which water was used in the entire wash-off process. This indicated that different rinsing mediums had compromised the integrity of fabric color consistency. Because fabrics showing a smaller Δ*E_cmc(2:1)_* have better color consistency with the reference fabric, F3_−r3_ and F4_−r3_ maintained higher integrity of color to F5_−r3_, if compared with F1_−r3_ and F2_−r3_. This was in line with the phenomenon demonstrated in Figure 10. Because the color density of E_r2−β_ was higher than that of E_r2−α_, the unfixed dyes on F3_−r3_ and F4_−r3_ had been stripped more extensively than those from F1_−r3_ and F2_−r3_. Color deviation was mainly caused by the adverse impacts from accumulated degradation products from catalytic ozonation of dyes. The presence of these organics in aqueous solution may have improved the substantivity/affinity of unfixed dyes on fabric. Thus the reverse rinsing step, in which the loose color/dyes were transferred from fabrics to effluents, was hindered, resulting in suppressed rinsing capacity of reclaimed effluents on unfixed dyes. Consequently, it was reasonable to assume that catalytic ozonation was more effective to recover the rinsing capacity of spent rinsing effluents. The presence of MnFe_2_O_4_@CA had significantly enhanced degradation efficiency of organics.

Furthermore, unlike F2_−r3_, which was cleaned with water in R3, lower color integrity was found in F1_−r3_. This indicated that water was still more favorable in stripping unfixed dyes from fabrics, which was compatible with the result that the absorbance of E_r3−α−w_ was about 35% higher than that of E_r3−α_ (Figure 9). However, the color difference between F2_−r3_ and F5_−r3_ was still apparent (Figure 11). This also showed that water in R3 could not improve the low-effective rinsing of fabrics in R2 because R2 was performed at 95 °C and was more capable than R3 to strip loose color. Although water could alleviate the ineffectiveness of fabric rinsing in R2 to some extent, in R3 it could not remove all unfixed dyes which should have been cleaned in R2. Thus water used in R3 still could not compensate for the decreased rinsing capacity of R2 when treated effluents were used.

In general the deviation of Δ*E_cmc(2:1)_* should be no more than 1.0; otherwise the human naked eye could identify the color inconsistency [48]. Thus, Δ*E_cmc(2:1)_* > 1 was a rejection criterion for color similarity. Both F3_−r3_ and F4_−r3_ were within the tolerance for color disconformity; on the contrary F1_−r3_ and F2_−r3_ exceeded the visible detection limit of human naked eye. Color reproducibility of RR239 and RY176 was similar to that of RB19. Consequently, it was convincible that the rinsing capacity of effluents could be recovered by catalytic ozonation as the resulting fabrics had good color similarity to the reference fabric. Conversely, ozonation alone may be not adequate to recover waste effluents because the resulting fabrics had inferior color similarity and met the rejection criteria of color reproducibility.

#### 2.3.2. Colorfastness

After dye fixation, wash-off is used as a cleaning treatment to remove unfixed dyes present on fabrics. It is an indispensable procedure to improve colorfastness of the final products. Thus, colorfastness was the most vital consideration for fabrics rinsed with recycled effluents. Colorfastness of typical fabrics dyed with three dyes are compared in Table 2. The fabric (F0) which was collected without dye fixation before rinsing was also included as a reference.

According to Table 2, F0 displayed the lowest colorfastness grades and was lower than F5_−r3_ by 1–2 grades. This offered compelling evidence that the colorfastness of fabrics not rinsed remarkably downgraded. Although colorfastness of F1_−r3_ and F2_−r3_ was higher than F0, it was still lower than that of the reference F5_−r3_ by at least 0.5 grade. This supported the deduction as shown in Figure 10 that there were some unfixed dyes still present over F1_−r2_ and F2_−r2_. By contrast, colorfastness of F3_−r3_ and F4_−r3_ was more similar to F5_−r3_. Equivalent colorfastness was obtained from both of these two fabrics, showing that catalytic ozonation was reliable for recycling spent effluents to maintain colorfastness.

#### 2.3.3. Color Evenness

Dyeing evenness is important in textile dyeing, but it is seldom evaluated because of the excellent migration properties of majority of the reactive dyes in exhaustion dyeing. However, it has to be considered during recycling spent effluents because some unfixed dyes exist in fabrics which resist rinsing with recycle effluents. The uneven distribution of loose color, which was from the unfixed dyes, could potentially influence the leveling performance of fabrics. Moreover, uncompleted oxidation products of dyes present in recycled effluents may also influence the adsorption, desorption and distribution behaviors of unfixed dyes on fabrics. Therefore, color evenness of fabrics treated by recycled effluents should be evaluated for color quality control.

**Figure 12:**
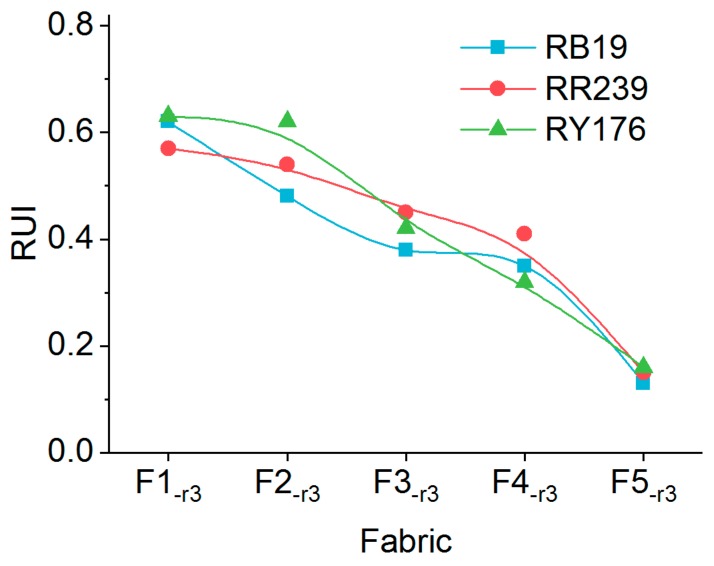
RUI of fabrics dyed with different dyes.

The leveling of color was measured by the relative unevenness index (RUI). There were four grades to classify leveling using RUI: excellent (RUI < 0.2), good (0.2 < RUI < 0.5), poor (0.5 < RUI < 1.0) and bad (RUI > 1.0). Figure 12 shows the RUI of fabrics treated with different rinsing mediums. Overall, RUI of fabrics treated with water was the smallest, corresponding to around 0.2 for all the three fabrics dyed with different colors, demonstrating that all the three dyes had excellent leveling property. The RUI of fabrics treated with recycled effluents was mostly higher than that of the reference fabric, confirming that the oxidation products of dyes fairly influenced the color evenness of fabrics. However, the leveling properties of F4_-r3_ were still acceptable because the RUI was no more than 0.5, although the recycled effluent affected the color evenness of fabrics.

## 3. Materials and Methods

### 3.1. Chemicals and Fabrics

C.I. Reactive Blue 19 (RB19), C.I. Reactive Red 239 (RR239), C.I. Reactive Yellow 176 (RY176), and bleached cotton knits (250 g/m^2^) without brightener was provided by Mansum Dyeing and Finishing Ltd. (Guangdong, China). Other chemicals used were of analytical grade and purchased from Millipore Sigma (Darmstadt, Germany).

### 3.2. Catalyst Preparation

The preparation approach of CA has been reported elsewhere [49]. The hydrothermal reaction was adopted to prepare MnFe_2_O_4_@CA. CA was crushed into fine particles with a miller (A11, IKA, Staufen, Germany) and then screened to isolate particles within the 80–100 mesh (150–180 μm) range. The fine particles were then dried in an oven at 102 °C for 2 h before weighing. After drying, 0.5 g CA was added into a Teflon-lined hydrothermal autoclave (100 mL) which was pre-filled with 60 mL of precursor solution (0.49 g FeCl_3_ and 0.19 g MnCl_2_·4H_2_O dissolved in 60 mL deionized water). The mixture was then magnetically stirred at room temperature for 20 min, and then 2 M NaOH solution (10 mL) was added dropwise. Subsequently, the reactor was kept at 120 °C for 8 h in an oven. Followed by natural cooling, the prepared magnetic particles were collected with a magnet and washed with absolute ethanol several times. Finally, the particles were dried at 80 °C for 4 h in an oven before further use. The pure MnFe_2_O_4_ was prepared following a similar approach, except in the absence of CA particles.

### 3.3. Experimental Procedure

#### 3.3.1. Fabric Dyeing

Wash-off experiment together with exhaustion and fixation of dyes were conducted using an infrared-heating dyeing machine. First, 5 g of cotton fabric was put into the dyeing vessel pre-filled with 100 mL of dyeing solution and containing 0.1 g dye and 4 g Na_2_SO_4_. Next, the dyeing vessel was loaded into the rotary dyeing machine, heated up to 60 °C at a rate of 2 °C/min, and were maintained at this temperature for 30 min for dye exhaustion of RB19. Afterwards, 1 g Na_2_CO_3_ was added into the dyeing vessels and was further maintained at 60 °C for 30 min to facilitate dye fixation on fabrics. Subsequently, dyed fabrics were squeezed by a horizontal textile padder to take with 80% (o.w.f) dyeing bath. The wet fabrics were then sealed in plastic bags separately and kept ready for rinsing in the wash-off step.

#### 3.3.2. Effluent Recycling in Wash-Off Process

**Figure 13:**
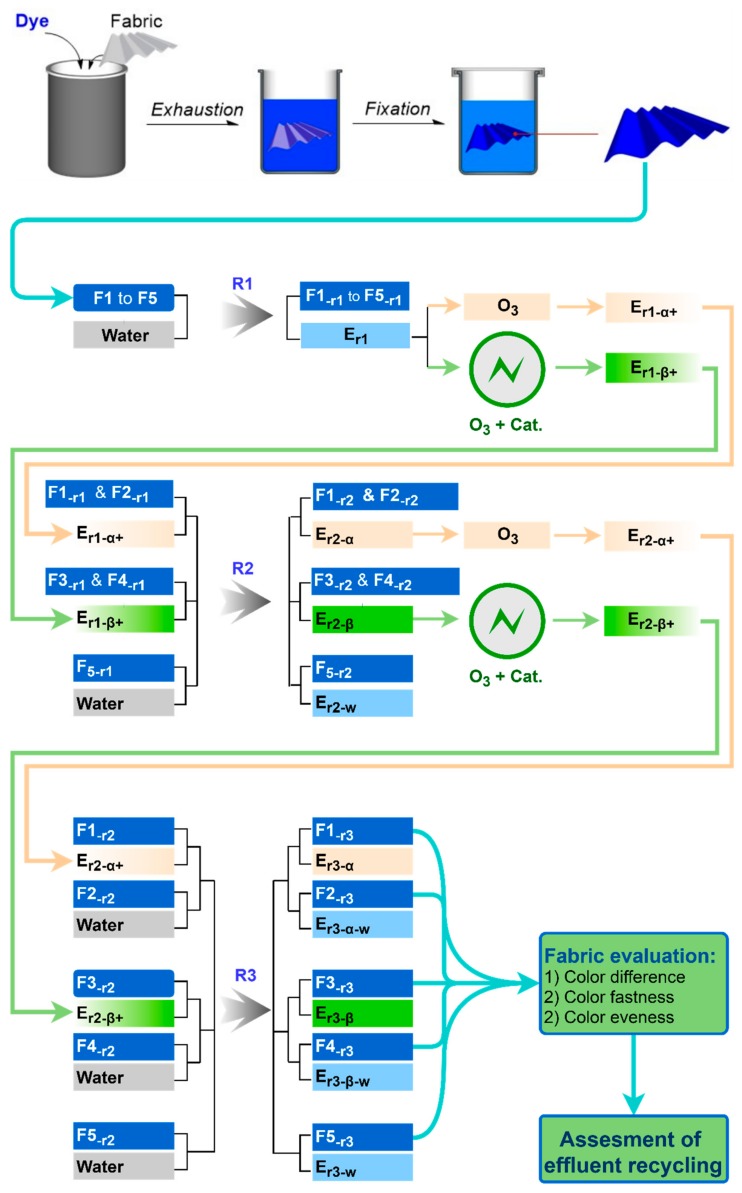
Flow chart of recycling waste rinsing effluents in wash-off process.

The wash-off process included three rinsing steps, R1, R2 and R3. R2 was performed at 95 °C, while both R1 and R3 were implemented at 60 °C. All the three steps lasted for 15 min. The different rinsing mediums, both water and treated effluents, used in the wash-off process are shown in Figure 13. There were total five groups of fabrics that were used, denoted as F1 to F5. Each group comprised three pieces of the same fabric that was dyed in a separated dyeing vessel. In R1, F1 to F5 were rinsed with water to form the first bath of the effluent (E_r1_), which was then treated by either ozonation alone (O_3_) or ozonation with catalyst (O_3_ + Cat.). After different treatments, E_r1_ was converted into treated effluents, E_r1−α+_ and E_r1−β+_; while F1 to F5 were transformed to F1_−r1_ to F5_−r1_. In R2, depending on the rinsing medium applied, the 5 groups of fabrics were divided into three categories: F1_−r1_ and F2_−r1_ were rinsed with E_r1−α+_ to obtain F1_−r2_ and F2_−r2_; similarly, F3_−r1_ and F4_−r1_ were rinsed with E_r1−β+_ to prepare F3_−r2_ and F4_−r2_. F5_−r1_ was cleaned with water as a reference. The collected waste effluents, E_r2−α_ and E_r2−β_, were then treated by ozonation or catalytic ozonation to form E_r2−α+_ and E_r2−β+_. Likewise, in R3, the 5 groups of fabrics were rinsed by five different rinsing media, and the resulting fabrics and effluents were collected for final property evaluation.

#### 3.3.3. Ozonation Treatment of Rinsing Effluents

Ozonation of rinsing effluents was performed in a glass cylinder equipped with a gas diffuser at the bottom of the reactor at room temperature. The ozone gas flow rate and concentration was set to be constant at 0.5 L/min and 8 mg/L. Before ozonation, the effluent was adjusted to pH 7.0. For each treatment, 0.5 g CA or MnFe_2_O_4_@CA, or 0.057 g MnFe_2_O_4_ was added into the cylinder before addition of 500 mL of effluents. Then the catalyst-effluent mixture was pre-stirred mechanically for 5 min to ascertain thorough mixing, before supplying ozone gas. Ozone gas was produced in-situ from oxygen using a precise ozone generator (Medozons BM-02, Nizhny Novgorod, Russia). After a predetermined time since the start of the treatment, 3 mL of effluent was sampled for determination of color and COD.

### 3.4. Analytical Method

We used X-ray diffraction (XRD) (SmartLab, Rigaku, The Woodlands, TX, USA), scanning electron microscope (SEM) (VEGA3, Tescan, Brno-Kohoutovice, Czech Republic), transmission electron microscope (TEM) (Model JEM-2100F, JEOL, Tokyo, Japan), Fourier-transform infrared spectroscopy (FTIR) (Spectrum 100, Perkin Elmer, Akron, OH, USA), thermal gravimetry analysis (TGA, TGA/DSC 1 Star System, Mettler Toledo, Toledo, OH, USA) to characterize the catalyst materials. The pore structure was measured by a surface texture analyzer (Nova Touch LX^4^, Quantachrome Instruments, Boynton Beach, FL, USA). Depending on the exact assays, the color density of effluents was measured by (1) either the maximum absorbance at *λ*_max_ = 593 nm, or (2) the absorbance ratio using Equation (1):

Color density = *A_t_*/*A*_0_ (1)

where, *A*_0_ is the absorbance of the original effluent without reclamation and *A_t_* is the effluent absorbance after ozonation treatment for time *t*.

Effluent COD was tested by the digestion method in accordance with the Hach Method 8000: Oxygen Demand, Chemical [50]. To ensure accuracy, each test was carried out three times to obtain an average value. Fabric color specifications, including reflectance, lightness and chromaticity were measured by a spectrophotometer (Datacolor Spectrum 650, Datacolor Inc., Lawrenceville, NJ, USA). Color difference (Δ*E_cmc(2:1)_*) was calculated according to equations described in the testing method of AATCC TM173-2013 [51]. The colorfastness of fabrics was evaluated in accordance with the standard testing methods AATCC TM61-2013 [52] and AATCC TM8-2016 [53]. In addition, a relative unlevelness index (RUI) was used to evaluate color leveling property, according to Equation (2) [54,55]:

(2) $$\mathrm{RUI}=\overset{700}{\underset{\lambda =390}{\sum }}\left(\frac{S_{\lambda }}{R}\right)V_{\lambda }$$

where *S_λ_* is the standard deviation of reflectance determined at the relative wavelength, *R* is the average value of reflectance, and *V_λ_* is the coefficients of variation of reflectance.

## 4. Conclusions

Recycling of wastewater is of growing interest in textile processing. However little attention has been paid to recycling spent rinsing effluents in the wash-off process. In the current study, catalytic ozonation was implemented to reuse spent effluents from the wash-off process by recycling spent effluent to alleviate water dependence. The results demonstrated that the magnetic catalyst used in this study significantly promoted dye degradation in the spent effluents. Compared with ozonation alone, the efficiency of dye degradation in terms of COD removal was remarkably enhanced by 25% by catalytic ozonation with MnFe_2_O_4_@CA. Accordingly, the enhanced efficiency of catalytic ozonation could allow the treated effluents to be recycled twice to clean loose color/dyes attached on fabrics. The final color quality of fabrics rinsed by treated effluents was hardly compromised and within the acceptable tolerance, when compared with that of the fabrics rinsed with clean water. Conversely, ozonation alone in the absence of MnFe_2_O_4_@CA could not achieve equivalent results under the same conditions. The resulting fabrics were of much poorer color quality and met the color reproducibility rejection criteria. By recycling the spent effluent produced via catalytic ozonation, the water consumption of the wash-off process could be remarkably reduced and the volume of wastewater discharged could be drastically decreased. These results conform to the trend of wastewater recycling in the textile industry. Moreover, our study may bridge the research gaps in recycling textile wastewater by catalytic ozonation, which is much easier to implement than other advanced oxidation processes.

## Figures and Tables

**Table 1:** Porosity and specific surface area of catalyst materials.

Materials	Pore Volume (cc/g)	Pore Radius (nm)	BET (m^2^/g)
MnFe_2_O_4_@CA 34.5%	0.81496	9.07018	378.565
MnFe_2_O_4_@CA 11.4%	0.99843	10.3588	467.092
CA	1.06902	8.76554	586.664

**Table 2:** Colorfastness of fabrics dyed with different dyes.

Dyes	Fabric	Laundering		Crocking	
		Staining (Cotton)	Change	Dry	Wet
RB19	F0	3.5	3	3	2
	F1_−r3_	4	3.5	3.5	3
	F2_−r3_	4	4	4	3.5
	F3_−r3_	4.5	4.5	4.5	4.5
	F4_−r3_	5	4.5	5	4.5
	F5_−r3_	4.5	5	5	4.5
RR239	F0	3.5	3	3	2
	F1_−r3_	3.5	3.5	3.5	2.5
	F2_−r3_	3.5	4	4	3
	F3_−r3_	4	4	4	3.5
	F4_−r3_	4.5	5	4.5	4
	F5_−r3_	5	4.5	4.5	4
RY176	F0	3.5	3	3	2.5
	F1_−r3_	4	3.5	3	3
	F2_−r3_	4.5	4	3.5	3.5
	F3_−r3_	4.5	4.5	4	4
	F4_−r3_	4.5	5	4.5	4
	F5_−r3_	5	4.5	4.5	4
